# Annotations

**Published:** 1891-04-11

**Authors:** 


### Annotations.

**The Abolition of Hypnotism.** It is seriously proposed to put an end, by law, to hypnotism, both as a method of medical treatment and as a stage performance. This is a proposal which we are strongly inclined to support. In The Hospital's Annual Summary for 1890, published on January 3rd, 1891, we wrote: "Hypnotism has greatly exercised the minds of both medical men and the public during the past year. It is flattering to our British strength of mind that fewer people in this country are found to be fit subjects for hypnotism than is the case in France; and it is a tribute both to the intelligence and the conscientiousness of British medical men that they regard the whole subject with wholesome suspicion . . . In our judgment, if hypnotism were never heard of more the world would not be the l ser . . . Hypnotism is no more to be practised except in cases of extreme necessity, than is chloralism, morphinism, or any other method of treatment of which the disadvantages are almost as great as the benefits. We congratulate ourselves and our fellow countrymen that our nerves are as yet so firm and steady that hypnotism is but a curiosity amongst us. Long may it remain so!" So far as we know we were the first journal to take that decided stand. It is eminently satisfactory to us to find that so cautious and influential a professional newspaper as the *British Medical Journal* is now inclined to stand on the platform of wholesome doubt and severe suspicion. The *Daily Graphic*, too, which, though not a professional journal, is conducted with much scientific discrimination, has had the sense to see the very doubtful advantages and the quite certain dangers of the practice of hypnotism. From a public and popular, but not from an ignorant standpoint, that newspaper calls for the abolition of hypnotism. In the *Daily Graphic* of Saturday, April 4th, a writer, signing himself "M.D. Lond.," says: "I trust your timely observations with regard to the abolition of hypnotism will be only the first step towards a speedy public recognition of the evils of this most dangerous practice. While such cases as that quoted from the *British Medical Journal* are undoubtedly rare, one such instance is quite sufficient to show the nerve-weakening effect of such foolhardy experiments. In my opinion the attack of acute mania which resulted on that occasion is far less serious, because temporary, than the permanent mental impairment of a slighter degree which is the commoner sequel, and which places the unfortunate individual within measurable distance of insanity. The marked loss of will-power which is engendered by such experiments is the first step towards imbecility, and is never, under the most favourable conditions, entirely recovered from. To men who have met such cases in practice the whole thing is very saddening. It seems easy enough to steal away a man's brains, but a very different matter to restore them. . . . Personally, I disbelieve entirely in the therapeutic value of all such experiments, even when conducted by the most careful and conscientious operators, and I know my opinion is shared by many medical brethren." This gentleman does well to have the courage of his convictions, and to express those convictions publicly. It is a pity he did not sign his name. There is a certain section of the public which holds that the medical profession is unduly conservative about new and peculiar remedies; and many medical men weakly yield to the popular feeling, lest they should be thought to be scientifically "behind the times." This is unworthy of men of science. Medical men are conservative about treatment for two reasons: First, because they have built up with infinite labour a system of experimental medicine which is based on the methods and ascertained facts of medical science; and, secondly, because they have found that those peculiar methods of treatment which cannot be shown to rest upon a basis of ascertainable physical facts are for the most part useless, and frequently also injurious. We are not among those who deny the right of intelligent laymen and of the public generally to inquire into the facts of medicine, or to offer their criticisms thereupon. On the contrary, we welcome and encourage all such inquiry and criticism. But when all possible freedom has been allowed to the laity, it still remains the fact that the intelligent and conscientious doctor is the man who really understands the business, and the man on whose proved results the ultimate decision must depend. Doctors suspect both hypnotism and hypnotists; and they do so justly. It is much to be desired in the public interests that they will express their convictions with greater boldness and openness, in order that they may take their true place as leaders, and not as followers, of the public judgment in matters medical.

**Science and Lynch Law.** The newspapers are still full of the lynching of certain Italians at New Orleans, some of whom were known assassins and others were suspected. Science has a comparatively short way of dealing with such cases. To her man is a social and gregarious animal. Men live together in societies because it is their nature to do so. Single individuals or pairs do not go about by themselves, as is the manner of some animals, except abnormally. But many persons can only live together in one society under certain well-defined conditions. The society must have regulations, and all the members of it must loyally submit to those regulations. There must be a recognised method of enforcing submission in cases of necessity; and if the recognised method fails, then the first efficient means that lie ready to hand must be put in force until a new regular method can be adopted and tried in practice. To science in the abstract those New Orleanists who put murderers to death by an irregular method of punishment because the regular method had failed, and because there was immediate urgent danger to the general well-being, simply did what all sorts and conditions of men, at every period of history and civilization, have done when the motive of necessity has been sufficiently strong to impel them. Not only so, but in the future, and in every part of the world, however much civilisation and culture may advance, the very same course of final appeal to the popular sense and the popular will may on occasions have to be resorted to. It is right that an inquiry should be demanded, and that a full investigation into all the facts should be made. It is probable that some of the lynched Italians have suffered unjustly. The families of those should be adequately compensated, and all possible reparation made. Those who have been proved to have been murderers have simply met with just retribution, and to compensate or make any sort of amends to their families would be both absurd and wicked. The leaders of the lynchers, so far from being worthy of punishment, deserve public praise as vindicators of elementary law and right. What compensation is ultimately needed must be made by the city and the state; and not only must such compensation be given to the families of the men who may have been lynched unjustly, but also to those leaders and vindicators of elementary public safety who may have suffered in purse or person for their readiness to stand, like Horatius, in the breach, when the liberty and life of their city was so wickedly threatened. Mere legal quibbles and technical points are entirely out of court in a case of this kind. The appeal must be to those broad facts and large rules which science has generalised for us when she has written the comparative history of man.

**Census Consciences.** There can be no manner of doubt that many persons understate their ages in the census returns. It is equally certain that most of these persons are women, and unmarried women. The writer knows of a particular cook who gave her age as thirty-six, though she cannot be less than forty-six; and of a certain housemaid who returned herself at twenty-eight, although she gave her age as twenty-eight three years ago when she entered upon her present situation. It is pretty certain that large numbers of women of all ranks have answered in a similar fashion, and perhaps a few men. The conclusion which forces itself upon the straightforward male mind is that women as a class have not the brains to estimate a lie at its proper value. A well-known novelist admits that women do not seem to be able to understand the nature and value of truth; and he defends them in their position. For our part, we look upon him as an enemy of women, and not as a friend, for so doing. Politeness may prompt him to occupy this peculiar ground; but politeness is politeness, and a lie is a lie. That a falsehood is morally bad nobody doubts; but it is also stupidly unscientific, and intellectually ridiculous. As a matter of polite breeding, it is entirely out of the running, for no liar can be a lady. We have purposely put the subject in a blunt, and perhaps even in a brutal light. If any apology be needed it is to be found in our high and sincere regard for the true nobility of character which is proper to the typical woman.

